# Assembly and comparative analysis of the complete mitochondrial genome of *Holmskioldia sanguinea*

**DOI:** 10.3389/fpls.2026.1867148

**Published:** 2026-06-16

**Authors:** Jianxin Xu, Xiaowei Mo, Jie Zhang, Ling Yuan, Guihong Xu

**Affiliations:** 1 School of Landscape and Ecology, Shenzhen City Polytechnic (Shenzhen Institute of Technology), Shenzhen, China; 2 Key Laboratory of Southern Subtropical Plant Diversity, Fairy Lake Botanical Garden, Shenzhen and Chinese Academy of Sciences, Shenzhen, China

**Keywords:** collinearity analysis, *Holmskioldia sanguinea*, mitochondrial genome, phylogenetic analysis, repeat sequence

## Abstract

**Introduction:**

*Holmskioldia sanguinea*, a distinctive ornamental plant native to the subtropical Himalayas, is valued for its unique hat-shaped calyces, strong adaptability, and ecological significance in slope restoration and nectar provision. Its phylogenetic position within Lamiales has remained controversial due to limited molecular evidence. Although earlier studies have successfully elucidated its chloroplast (cp) genome, the complete mitochondrial (mt) genome remains uncovered.

**Methods:**

This study undertook the sequencing, assembly, and comprehensive analysis of the complete mt genome of *H. sanguinea*.

**Results:**

The mt genome of *H. sanguinea* is a circular DNA molecule measuring 405,461 bp in length with a GC content of 46.00%. It contains 36 protein−coding genes (PCGs), three ribosomal RNAs (*rrn5*, *rrn18*, *rrn26*) and 33 transfer RNAs. The mt genome is rich in repetitive sequences; a total of 550 interspersed repeats were identified, including 300 palindromic repeats (54.55%) and 250 forward repeats (45.45%), without inverted or complementary repeats. Additionally, 79 simple sequence repeats (SSRs) were detected, with tetranucleotide repeats (AAAG/CTTT) being the most abundant. Codon usage analysis revealed a preference for A/U−ending codons, and the codon GCU (Ala) exhibited the highest relative synonymous codon usage (RSCU) value of 1.57. A total of 565 RNA editing sites (all C−to−U conversions) were predicted, with the majority (80.71%) potentially leading to increased protein hydrophobicity. Six cp plastid DNAs were identified in the mt genome, indicating intracellular gene transfer events. Most mt genes are under strong purifying selection (Ka/Ks < 1), while nucleotide diversity analysis showed that *atp9* had the highest diversity (Pi = 0.06444) and *nad4L* the lowest (Pi = 0.00305). Phylogenetic analysis based on 14 shared mt PCGs placed *H. sanguinea* within Lamiaceae, forming a well-supported clade with *Lavandula angustifolia*, *Salvia officinalis*, *Elsholtzia blanda*, *Callicarpa nudiflora* and *Vitex trifolia* (bootstrap = 100%). Collinearity analysis revealed extensive genome rearrangements among related species, and gene content analysis indicated the loss of *rps7*, *rpl2* and *sdh3* as well as duplications of *atp1*, *cox1* and *nad4*.

**Discussion:**

Understanding the mt genome characteristics of *H. sanguinea* is critical for elucidating its phylogenetic placement and genetic background. The results of this study serve as a foundation for future genetic, evolutionary, and breeding studies of this ornamental and potentially medicinal species.

## 1 Introduction

*Holmskioldia sanguinea* is a distinctive ornamental plant native to the subtropical regions of the Himalayas, known for its striking reddish-brown inflorescences and unique hat-shaped calyces (Atkins, 1996). Since its introduction to the Royal Botanic Gardens, Kew in 1795 by plant collectors Peter Good and Christopher Smith (Lee and Kim, 2020), it has been widely cultivated across southern Asia, Mauritius, Indonesia, and the West Indies, where it has become naturalized in many regions. Due to its strong adaptability, ease of propagation, and ornamental value, *H. sanguinea* is also commonly used in slope restoration and ecological landscaping (Xu et al., 2023). Additionally, it serves as an excellent nectar source, effectively attracting birds and butterflies (Atkins, 1996; Lin et al., 2017). Extracts from its aerial parts, containing compounds such as oroxindin, have shown significant antifungal activity against pathogens like *Alternaria alternata* and *Fusarium fusiformis* (Chaudhuri et al., 2004), indicating its potential medicinal value.

Traditionally, the genus *Holmskioldia* belongs to the family Verbenaceae (Ryding, 1995). However, recent molecular phylogenetic studies using nuclear and chloroplast markers suggest that *Holmskioldia* belongs to Lamiaceae, which is sister to *Scutellaria* L (Li et al., 2016; Lee and Kim, 2020). They share the features of rounded calyx lobes (at anthesis) and a pericarp surface which has ridges and other characteristics in common. Three plants within the *Holmskioldia* genus were moved to the genus *Karolia* (Atkins, 1996; Li et al., 2016; Lee and Kim, 2020). Thus, *H. sanguinea* belongs to *Holmskioldia* L., which is a monotypic genus. However, its phylogenetic position has remained controversial (Li et al., 2016; Lee and Kim, 2020), and precise classification of *H. sanguinea* requires additional molecular evidence.

Although both mitochondrial (mt) and chloroplast (cp) genomes are maternally inherited in plants, they differ significantly in structure, evolutionary rate, and recombination frequency (Zervas et al., 2019). The mt genome generally exhibits a slower nucleotide substitution rate but undergoes more frequent structural rearrangements and often incorporates sequences transferred from the cp genome. These characteristics make the mt genome a valuable molecular marker for studying species origins, phylogenetics, and population genetic diversity (Liu et al., 2025). Compared to nuclear and chloroplast genomes, the mt genome offers unique advantages in resolving deep phylogenetic relationships, revealing horizontal gene transfer events, and investigating mechanisms of adaptive evolution (Fonseca et al., 2008; Kong et al., 2025).

Mt genomes have been widely used to reconstruct phylogenetic relationships, shedding light on species’ geographical distributions and migration histories (Arseneau et al., 2017). The analysis of mt genome sequences is crucial for comprehending the evolution of various plant species (Wang et al., 2024b). In this study, we assembled and annotated the mt genome of *H. sanguinea* from the genus *Holmskioldia* (Lamiaceae), and further analyzed key genomic features such as codon usage, repetitive sequences, and mt plastid DNAs (MTPTs), as well as its phylogenetic position. Moreover, the findings are expected to provide important insights into the evolutionary history, taxonomic placement, and genetic basis of adaptation of *H. sanguinea*, while also contributing to comparative and evolutionary studies of mt genomes in Lamiales.

## 2 Materials and methods

### 2.1 Plant materials sampling, DNA extraction, and sequencing

The young and healthy leaves of *H. sanguinea* were harvested from Shenzhen Fairy Lake Botanical Garden, located in Luohu district, Shenzhen, Guangdong (coordinates: 114°10′41”E, 22°35′0”N). Immediately after collection, the samples were flash-frozen in liquid nitrogen and then stored at -80 °C for further processing. To extract genomic DNA, the Plant DNAzol Reagent (Invitrogen) was utilized according to the manufacturer’s guidelines. The agarose gel electrophoresis and a NanoDrop spectrophotometer (Thermo Fisher Scientific) were used to evaluate the quality and concentration of the extracted DNA. A 15 kb library was constructed using a SMRTbell Express Template Prep Kit 2.0 (Pacific Biosciences, CA, USA). The construction included DNA shearing, AMPure PB bead purification, ssDNA overhang removal, damage repair, end repair, hairpin adapter ligation, and library bead purification. Following quality control, a SMRTbell library was obtained. The library was sequenced on the PacBio Revio platform (Pacific Biosciences, CA, USA) by Shenzhen Huitong Biotechnology Co., Ltd. The CCS algorithm (version 6.0.0) was employed to process the raw data. To generate highly accurate HiFi reads, the parameters were set as follows: -minPasses 3, -minPredictedAccuracy 0.99, and -maxLength 21,000.

### 2.2 Mitochondrial genome assembly and annotation

The PacBio HiFi reads with total sequencing data size >6 Gb and average read length >17 kb were assembled using PMAT2 (v2.0.1) (Bi et al., 2024), a pipeline specifically designed for complex plant mt genomes. Assembly graphs were visualized and manually curated using Bandage (v0.8.1) to generate a preliminary mitochondrial genome assembly (Wick et al., 2015). The HiFi reads were then mapped to the assembled sequences with minimap2 (v2.24) (Li, 2018), and the draft assembly was further polished using NextPolish (v1.3.1) followed by manual adjustment to produce the final mt genome sequence and corresponding assembly graphs. The complete mt genome of *H. sanguinea* was annotated using MITOFY (Alverson et al., 2010) and MFANNOT (Beck and Lang, 2010), and a genomic map was generated using OGDRAW (Greiner et al., 2019).

### 2.3 Analysis of repeat sequences

Simple Sequence Repeats (SSRs) were identified with the MISA v1.0 tool (Thiel et al., 2003), with the parameters set to 1-10, 2-5, 3-4, 4-3, 5-3 and 6-3. Tandem repeat sequences were detected by TRF software (version trf409. linux64) (Benson, 1999), employing the parameters: match = 2, mismatch = 7, indel = 7, match probability = 80, indel probability = 10, minimum alignment score = 50, maximum period size = 2000, with additional flags -f -d -m. Interspersed repeated sequences were analyzed using REPuter v2.74 (Kurtz et al., 2001), an online tool available at https://bibiserv.cebitec.uni-bielefeld.de/reputer/; during the analysis, the minimum repeat size was set to 30 bp, and the sequence identity was set to 90% (i.e., the Hamming distance was set to 3). All identification results were visualized using Circos v0.69-8 (Zhang et al., 2013).

### 2.4 Codon usage bias analysis

To ensure the accuracy of the results, duplicate sequences and sequences with a length of less than 300 bp were excluded; sequences starting with the initiation codon ATG and terminating with the termination codons TAA, TAG, or TGA were selected. Subsequently, the standard protein-coding sequences (CDSs) were input into CodonW v1.4.4 software (http://codonw.sourceforge.net) in FASTA format for codon bias analysis, and the relative synonymous codon usage (RSCU) values were calculated. Histograms were plotted using R v4.5.1 (ggplot2 package; Wickham, 2009).

### 2.5 Gene transfer analysis

Homology searches were performed between the mt and cp genomes of *H. sanguinea* using Blastn v2.9.0+ (Altschul et al., 1990) with the parameters set to -evalue 1e-5 and -word_size 7. Only fragment transfer events with an alignment length exceeding 1000 bp were focused on to identify transferred genes between the two genomes. Short fragments were excluded to avoid false−positive homoplastic signals and random sequence noise, ensuring high confidence in MTPT identification. The results of the homology searches were visualized using Circos v0.69-8 (Zhang et al., 2013).

### 2.6 RNA editing site prediction

RNA editing events in the mt genome of *H. sanguinea* were predicted using the PREP-Mt program (http://prep.unl.edu/), with a cutoff value set to 0.2. Prior to prediction, the standard protein-coding sequences (CDSs) of the mt genome were extracted and formatted into FASTA format as the input data, ensuring no missing bases or frame-shift mutations in the sequences. The PmtREP platform was then accessed, and the FASTA-formatted CDS sequences were uploaded to the platform according to the operation guidelines. The prediction was performed using the platform’s default parameters optimized for plant organellar RNA editing, which mainly targets C-to-U editing events (the most common type of RNA editing in plant mitochondria).

### 2.7 Nucleotide diversity (Pi) analysis

Orthofinder software (v2.5.5) was used to identify the homologous core genes among 12 comparative species. The program was run with parameters -t 16 (16 threads), -f faa/(input protein directory), and -S blast (BLAST homology search). and a total of 26 common homologous core genes (**Supplementary Table 9**). These 12 species included *H. sanguinea* and 11 other species (*Elsholtzia blanda, Lavandula angustifolia, Salvia officinalis, Vitex trifolia, Callicarpa nudiflora, Avicennia marina, Strobilanthes sarcorrhiza, Sesamum indicum, Pedicularis chinensis, Triphysaria versicolor, Verbena officinalis*), whose mt genomes have been deposited in the GenBank of NCBI. Subsequently, the homologous gene sequences from different species were subjected to global alignment using MAFFT software (v7.525) with default settings. The aligned sequences were then input into DnaSP6 software (v6.12.03) to calculate the Pi value (nucleotide diversity) of each gene (Rozas et al., 2017), with the window length set to 200 bp and the step size set to 100 bp. Finally, the calculated Pi values were visualized for subsequent analysis.

### 2.8 Selective pressure calculation (Ka/Ks)

The acquisition of homologous core genes among 12 comparative species was performed using the same method as that for Pi value analysis: Orthofinder software (v2.5.5) was used to identify 26 common genes (**Supplementary Table 9**) from the 12 species (including *E. blanda, L. angustifolia*, et al. and *H. sanguinea*). The program was run with parameters -t 16 (16 threads), -f faa/(input protein directory), and -S blast (BLAST homology search). The 12 species were paired pairwise to extract homologous gene pairs, which were aligned using Blastn v2.9.0+ (-evalue 1e-5, -max_target_seqs 1) to retain optimal matches. After merging target and reference species sequences, ParaAT v2.0 software (default parameters) was used for formatting. Ka and Ks values of each gene pair were calculated by KaKs_Calculator v2.0 (https://sourceforge.net/projects/kakscalculator2/) with the MLWL method (Zhang et al., 2006), and Ka/Ks values were counted and visualized as box plots using R v4.5.1 (ggplot2 package; Wickham, 2009).

### 2.9 Collinearity analysis

Homologous sequences among the mt genomes of 12 related species (including *E. blanda, L. angustifolia, S. officinalis, V. trifolia, C. nudiflora, A. marina, S. sarcorrhiza, S. indicum, P. chinensis, T. versicolor, V. officinalis*, and *H. sanguinea*) were identified using Blastn v2.9.0+ (Altschul et al., 1990) with parameters set to -evalue 1e-5 and -word_size 7. Homologous sequences with an alignment length exceeding 1000 bp were selected as conserved syntenic blocks to construct a Multiple Synteny Plot, which was visualized using TBtools v2.467 (Chen et al., 2020).

### 2.10 Phylogenetic analysis

Orthofinder software (v2.5.5) (Kalyaanamoorthy et al., 2017) was used to identify homologous single-copy core genes among 23 comparative species for both chloroplast and mt genomes: a total of 49 homologous single-copy core genes were obtained from the cp genomes (**Supplementary Table 10**), and 14 homologous single-copy core genes were obtained from the mt genomes (**Supplementary Table 10**). The program was run with parameters -t 16 (16 threads), -f faa/(input protein directory), and -S blast (BLAST homology search). Multiple sequence alignment was performed using MAFFT software (v7.525) (Zhang et al., 2020) with the --auto strategy. Alignment was conducted based on the codon mode, with Codon Table 11 selected for chloroplast sequences and Codon Table 1 selected for mt sequences. Subsequently, the alignments were filtered with Gblocks 0.91b (Talavera and Castresana, 2007) with the parameter -t=c specified for codon−based sequence filtering and all other parameters set to default values to retain conserved alignment blocks. The filtered sequences were concatenated for phylogenetic tree construction. Maximum Likelihood (ML) tree inference was performed using IQ-TREE software (v3.0.1) (Ronquist et al., 2012). The ModelFinder tool integrated in IQ-TREE was used to select the optimal nucleotide substitution model based on the Bayesian Information Criterion (BIC) (Stamatakis, 2006): the optimal model for chloroplast sequences was GTR+F+R3, and that for mitochondrial sequences was GTR+F+I+R2. The number of bootstrap replicates was set to 1000. *Chionanthus retusus, Jasminum sambac* and *Forsythia ovata* were designated as the outgroup taxa.

## 3 Results

### 3.1 Genomic characteristics of the mt genome in *H. sanguinea*

**Figure 1:**
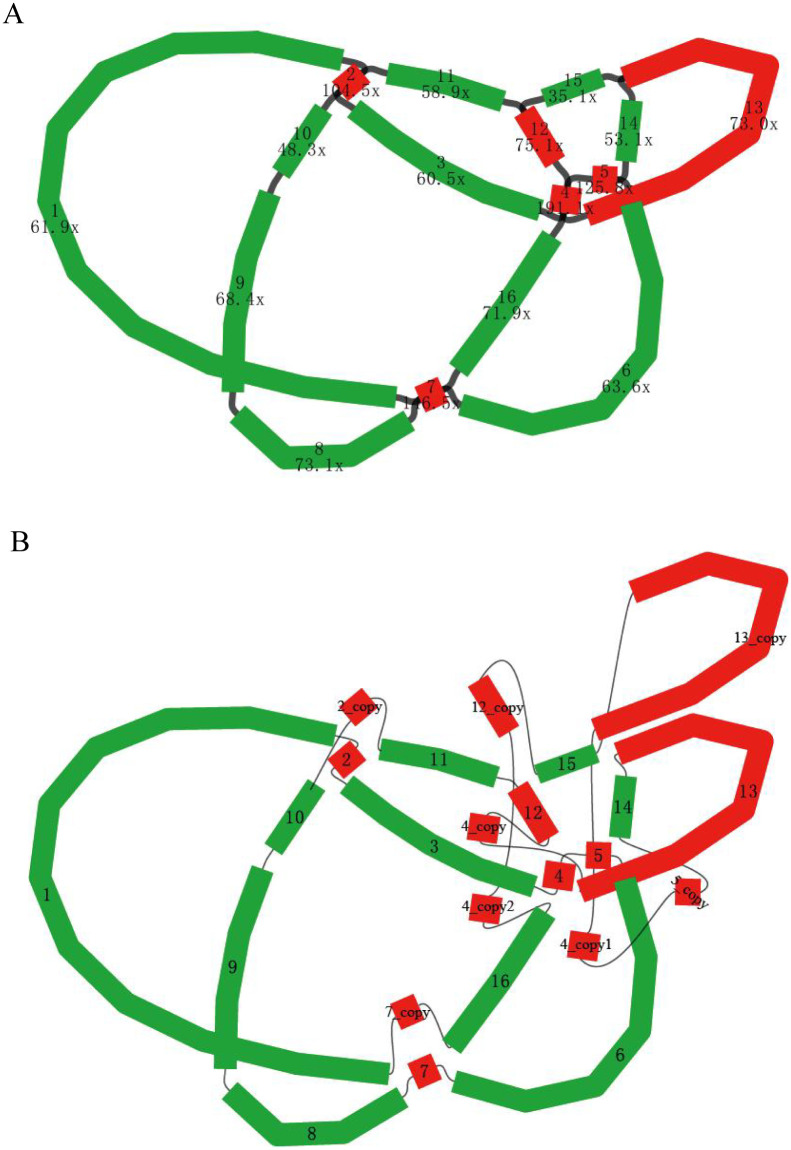
Genome assembly graph of the *H. sanguinea* mt genome. **(A)** The basic conformation of the *H. sanguinea* mt genome consists of a complex closed structure (multi-branched conformation), including 16 nodes. These nodes are connected to each other by lines, and the red nodes represent the predicted repeat areas. **(B)** Based on the sequential connection order of nodes, a schematic diagram of the linearization and recircularization process of the mt multi-branch structure.

**Figure 2:**
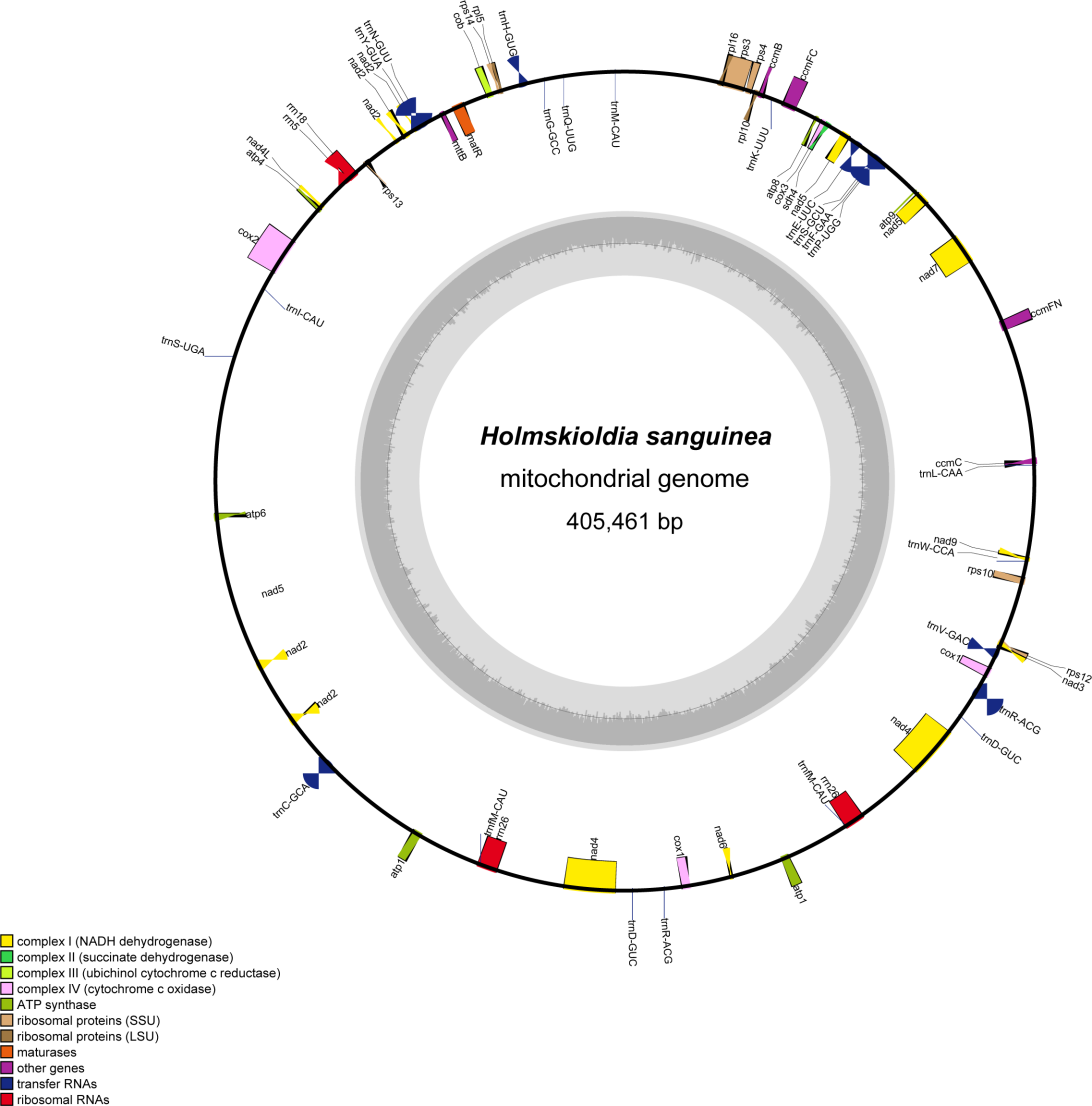
In the annotated map of the *H. sanguinea* mt genome structure. The genes in the ring represent the counterclockwise direction of transcription, while the genes outside the ring are in the opposite direction. Different functional genes are marked with different colors. The built-in gray histogram shows the genomic GC content, and the middle gray line is the 50% threshold line.

The complete mt genome of *H. sanguinea* exhibited a multi-branched conformation (**Figure 1A**), which contained 16 nodes. This **Figure 1A** generated from PacBio HiFi sequencing data and visualized using Bandage software, annotates each sequence fragment with node numbers and sequencing coverage depth. Nodes are interconnected by sequences, with red nodes representing predicted repeat regions that may mediate recombination of the mt genome. For subsequent analysis, we unlooped and integrated the multi-branch closed structure consisting of 16 nodes into a single circular contig, assembled in the following node connection order: 1→2→3→4→5→6→7→8→9→10→2→11→12→4→13→14→5→4→13→15→12→4→16→7→1. Long-read mapping across repeat regions (**Supplementary Figure 1**) confirmed that multiple conformations are supported by the sequencing data. Coverage across the entire assembly was consistent (mean depth 71.13×, no region below 50×, indicating that no major conformation is under-represented. This yielded a complete circular mt genome with a total length of 405,461 bp (**Figures 1**, **2**, **Supplementary Table 1**). The GC content was 46.00%, and the other base contents were: A 27.13%, C 23.01%, G 22.98%, and T 26.87%. In the genome, the CDSs covers 34,680 bp, with a GC content of 42.25% and an AT content of 57.75%. The genetic sequence of transfer RNA (tRNA) was 1,728 bp in length, with a GC content of 51.57% and an AT content of 48.43%. The genetic sequence of rRNA was 8,627 bp, which has 52.03% GC and 47.97% AT. The gene encoding the most amino acids is *nad5* (670 AA), and the gene encoding the least amino acids is *atp9* (74 AA). These results indicate that protein CDSs have a lower GC content than tRNA genes.

The mt genes of *H. sanguinea* can be divided into 10 categories, among which NADH dehydrogenase genes are the most abundant, including 9 genes. Genes containing four introns are *nad1*, *nad2*, and *nad5*; genes containing three introns are *nad7* and *nad4*; genes containing one intron are *ccmFc*, *cox2*, *rps10*, and *rps3*; and the remaining genes do not contain introns (**Supplementary Table 2**).

### 3.2 Repeated sequences in the *H. sanguinea* mt genome

**Figure 3:**
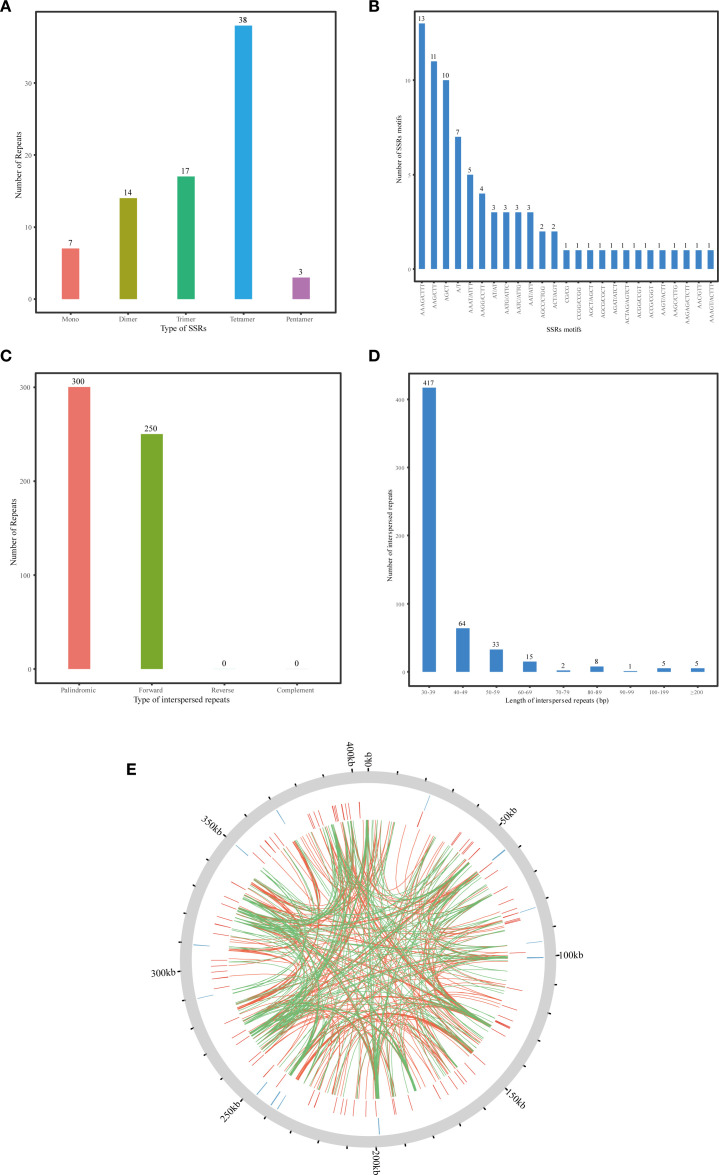
**(A)** Distribution of SSR in the *H. sanguinea* mt genome. The X-axis represents the types of SSR, while the Y-axis shows the quantity of SSR. **(B)** Distribution of SSR motifs in the *H. sanguinea* mt genome. The X-axis represents the types of SSR motifs, while the Y-axis shows the quantity of SSR motifs. **(C)** Distribution of interspersed repeat in the *H. sanguinea* mt genome. The X-axis represents the types of interspersed repeat, while the Y-axis shows the quantity of interspersed repeat. **(D)** Distribution of interspersed repeat lengths in the *H. sanguinea* mt genome. The X-axis represents the types of interspersed repeat lengths, while the Y-axis shows the quantity of interspersed repeat lengths. **(E)** Distribution of repeated sequence in the *H. sanguinea* mt genome. The green lines on the innermost circle connect the Forward repeats with interspersed repeat, and the red lines connect the Palindrome repeats with interspersed repeat. The red line on the second circle represents a SSR, and the blue line on the outermost circle represents a tandem repeat.

SSRs, also known as microsatellite DNA, are tandemly repeated sequences of 1-6 bp in length, which play a crucial role in genome variation, evolution, stability and organization. In this study, we found a total of 79 SSRs from the intergenic spacer (IGS), intron region, and coding region of the mt genome of *H. sanguinea*, with numbers of 58, 11, and 10, respectively (**Supplementary Table 3**). Specifically, mononucleotide repeats accounted for 8.86%, dinucleotide repeats for 17.72%, trinucleotide repeats for 21.52%, tetranucleotide repeats for 48.10%, and pentanucleotide repeats for 3.82% (**Figure 3A**). No hexanucleotide repeats were identified. The tetranucleotide repeats of AAAG/CTTT were identified as the most abundant SSR types, with a total of 13, accounting for 34.21% of the tetrameric SSRs, followed by the trinucleotide repeats of AAG/CTT, with a total of 11 (**Figure 3B**). The abundance of SSRs provides a rich resource for the development of molecular markers that are valuable for the identification and genetic characterization of *H. sanguinea*.

Tandem repeats, also known as satellite DNA, are repeated many times in tandem. They are widely distributed in eukaryotic genomes and prokaryotes. In this study, 15 tandem repeats were identified (**Supplementary Table 3**). The base length of these repeat units ranged from 13 bp to 70 bp, and repeats of longer segments (41–70 bp) were dominant, with a total of seven repeats. The number of copies of all duplications ranged from 1.9 to 2.8, indicating that most of the duplications were low-copy tandem duplications. Sequence similarity (percentage of match) was high, ranging from 80% to 100%, with seven sequences matching more than 90%, including two identical (100% match) repeat units. It is worth noting that some sequences (such as sequence numbers 12 and 14, 13 and 15) have the same repetitive unit sequence, but they are located in different regions of the genome, which may belong to the same kind of repetitive elements in different regions. On the whole, these tandem repeats are conserved in the genome, and the longer repeat units and higher sequence similarity may imply that they have certain functional or structural significance.

In addition, the interspersed repeats in the mt genome of *H. sanguinea* were analyzed, and 550 pairs of repetitive sequences with a length of greater than or equal to 30 bp were found in the mt genome, of which 250 pairs were forward matches, 300 pairs were palindromic matches, and reverse matches and complement matches were not detected (**Figure 3C**). Forward and palindromic duplications accounted for 45.45% and 54.55%, respectively. Specifically, approximately 96.2% of the repeats were between 30 and 69 bp in length, with the largest number of repeats in the 30–39 bp range, containing 417 repeats, compared to the smallest number of repeats in the 90–99 bp range, containing only one repeat (**Figure 3D**). Subsequently, in order to show the distribution of repetitive sequences on the mitochondrial genome of *H. sanguinea* more intuitively, we drew a loop diagram of repetitive sequences (**Figure 3E**). The analysis of these repetitive sequences provides direct clues to understand the structural dynamics and potential functional significance of the *H. sanguinea* mt genome, thus enhancing the understanding of the mechanisms of genome regulation and evolution in this species.

### 3.3 Codon usage bias in PCGs

**Figure 4:**
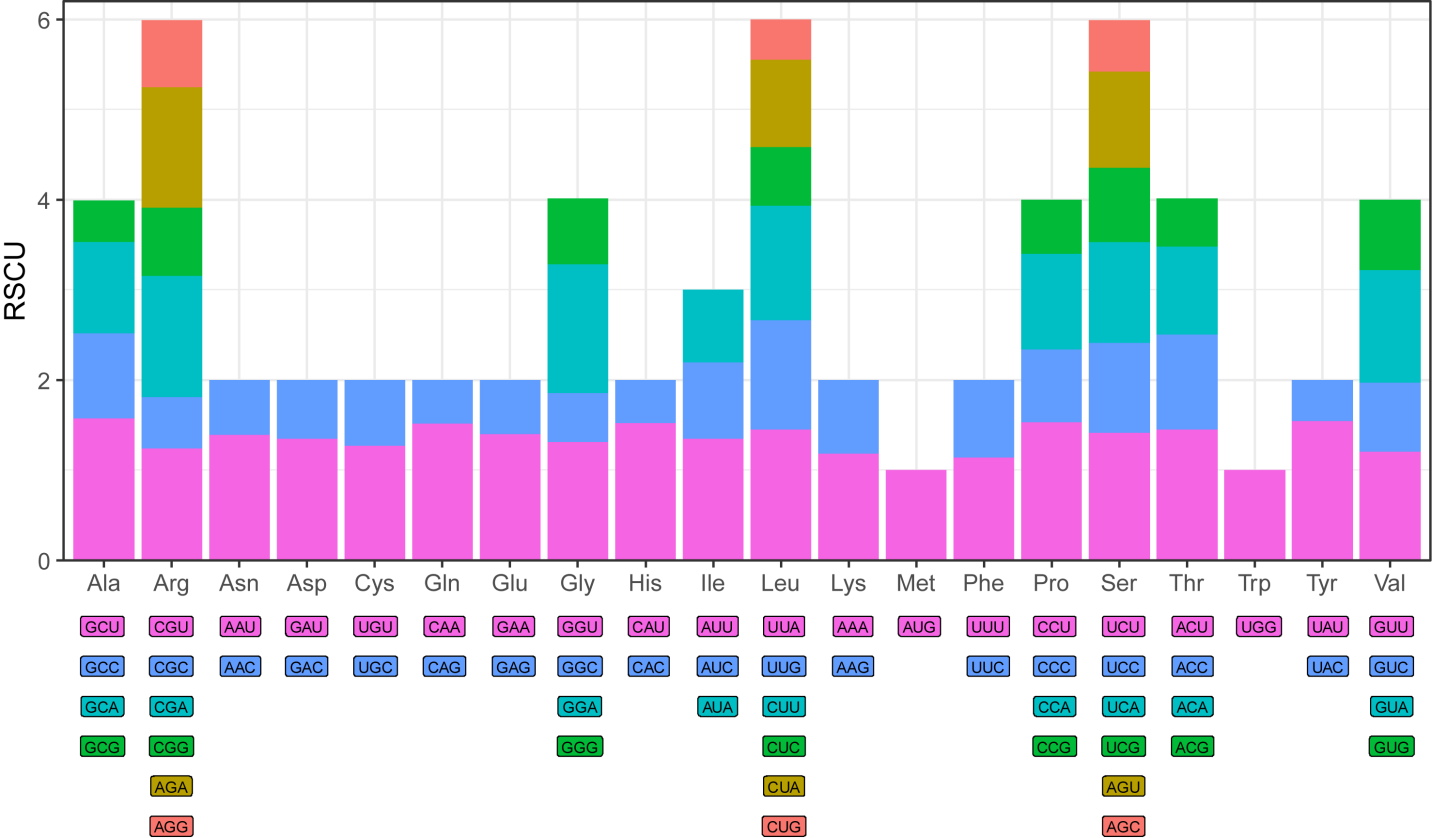
Relative synonymous codon usage (RSCU) of *H. sanguinea* mt genome. Codon families are on the X-axis. RSCU values are the number of times of a particular codon.

28 PCGs (**Supplementary Table 4**) were analyzed, and the codon usage of each amino acid is shown in **Supplementary Table 5**. Codons with an RSCU greater than 1 are considered to be used with amino acid preference. As shown in **Figure 4**, there is also a general codon usage preference for mitochondrial PCGs, except that the RSCU values of the start codon (AUG), tryptophan (UGG) and serine (UCC) are all 1. When RSCU > 1, the mitochondria contained 5,291 codons, indicating that the mt genes of *H. sanguinea* preferred to use these codons. Among these high-frequency codons (RSCU > 1), the third codon position is A or U (except ACC and UUG). Among the low frequency codons (RSCU < 1), the third codon position is mostly G or C. This is a common feature of codon bias in the organelle genomes of terrestrial plants. Alanine (Ala) in the mitochondria of *H. sanguinea* had a preference for the GCU codon, and its RSCU value was the highest among mitochondrial PCGs, reaching 1.57. Within amino acids, the usage of different codons varies significantly; for example, leucine prefers UUA and UUG, while arginine prefers AGA and CGU. In addition, UAA in the stop codon was used with obvious preference. These results suggest that this mt genome has a distinct non-random pattern in codon usage, possibly reflecting its adaptive strategies in translation efficiency or genome evolution.

### 3.4 Prediction of RNA editing sites

**Figure 5:**
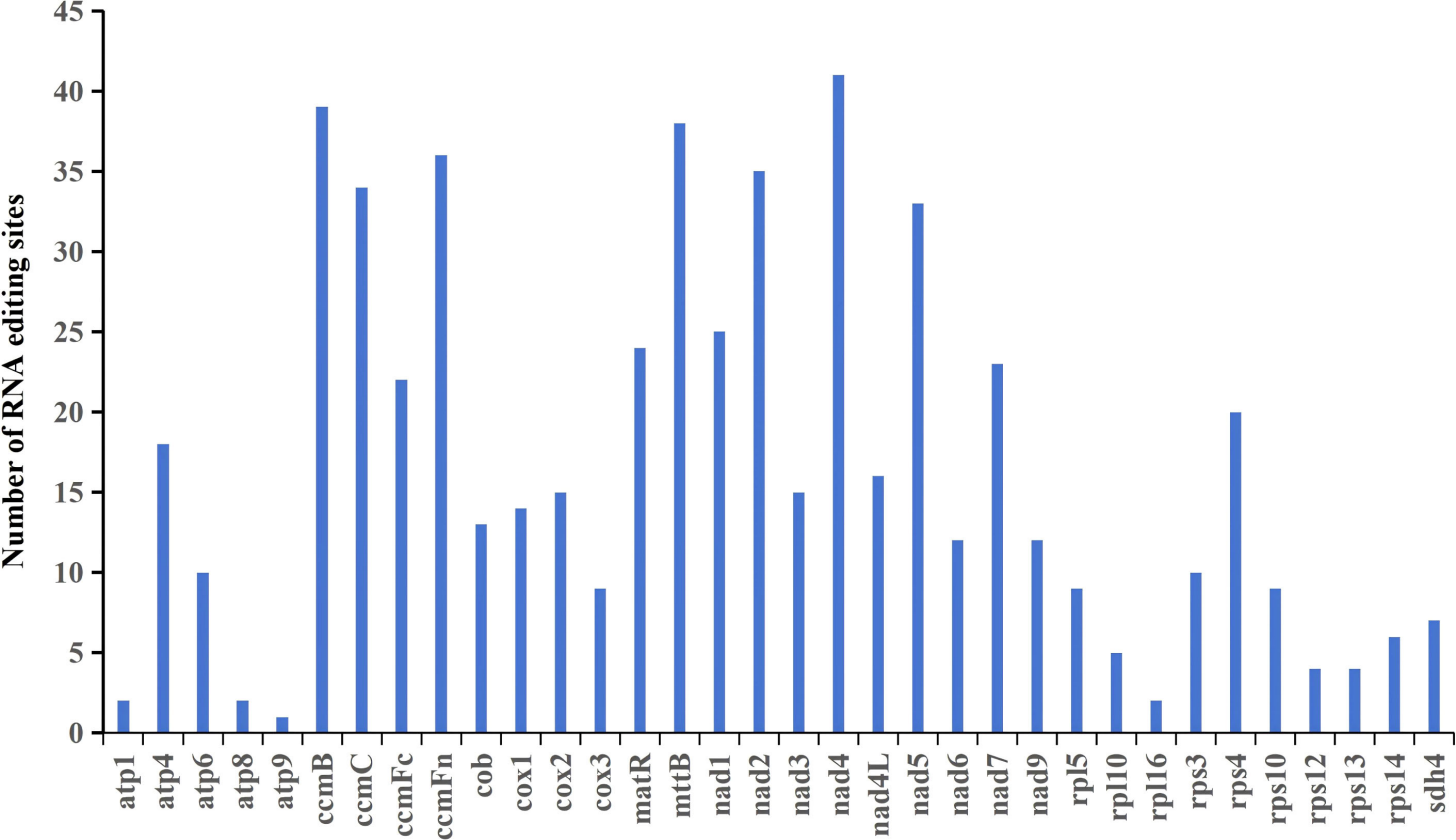
Predicted number of RNA editing sites for each PCG.

The prediction results of RNA editing sites of 34 protein-coding genes in the *H. sanguinea* mt genome show that RNA editing is common and has obvious characteristics. A total of 565 editing sites were predicted in 34 genes, but there were significant differences in the number of editing sites among different genes: *nad4* had the most editing sites (41), while *atp9* had the least (only one). The distribution of the number of editing sites (**Figure 5**) shows this imbalance.

All predicted editing events are C-to-U transitions (i.e., the C of the genome becomes U in the RNA after transcription) (**Supplementary Table 6**). These editing events can be divided into three categories according to the changes in amino acid properties they cause: most of the edits (456, accounting for 80.71%) resulted in the change of amino acids from hydrophilic to hydrophobic, including serine (S) to leucine (L) or phenylalanine (F), and proline (P) to leucine (L). Another 108 edits (19.12%) occurred between hydrophilic amino acids, such as histidine (H) to tyrosine (Y); only one edit was found to result in the generation of a stop codon (**Supplementary Table 6**). These results suggest that RNA editing in *H. sanguinea* mitochondria mainly tends to increase the hydrophobicity of the encoded proteins. These editing events are typical of plant mitochondria and predominantly restore hydrophobic residues that are important for membrane protein function.

### 3.5 Chloroplast-originated sequences in the mt genome

**Figure 6:**
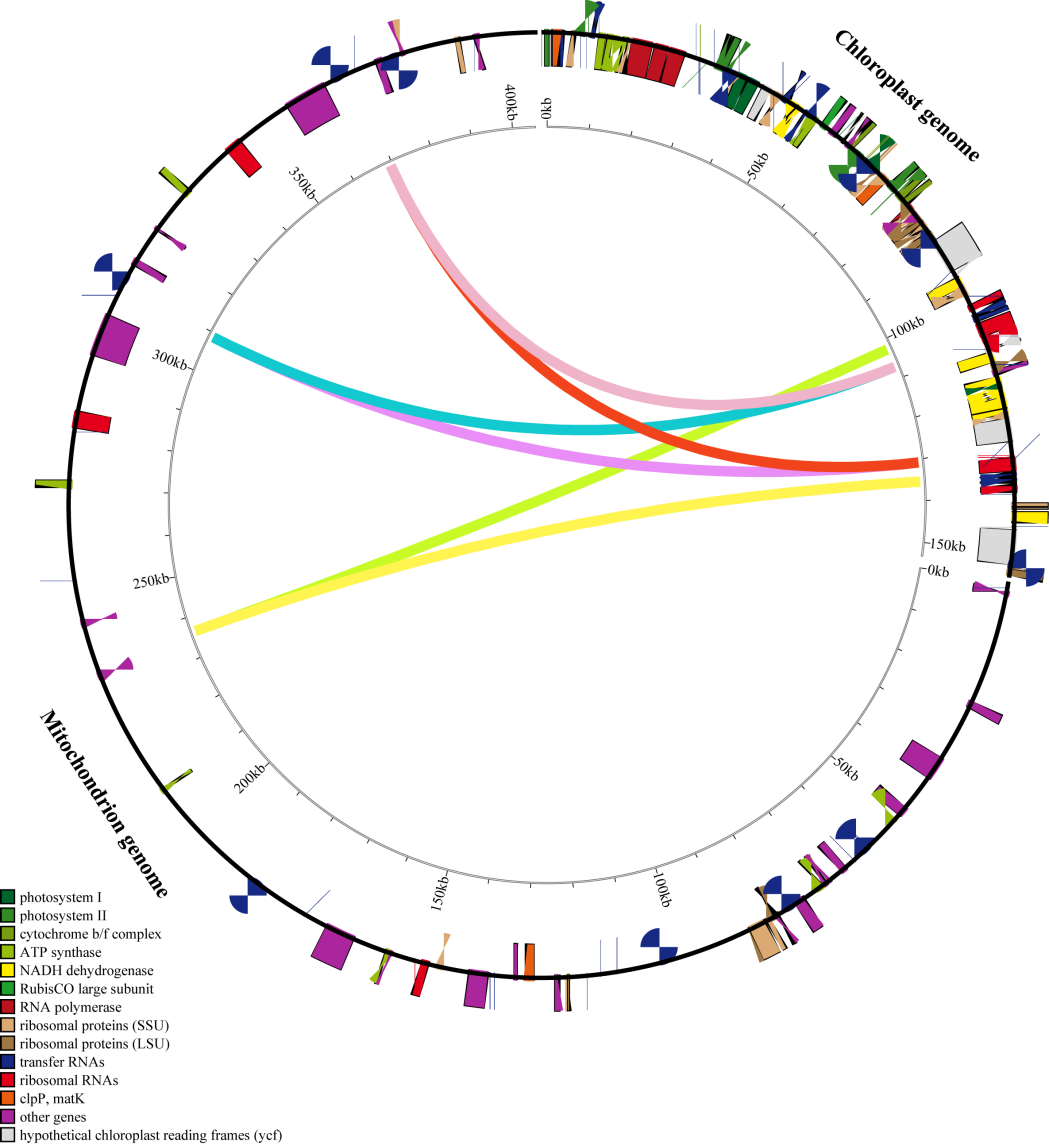
Analysis of sequence migration between cp and mt genomes in *H. sanguinea*. The genome segment corresponding to the connecting line between the arcs in the figure is a homologous segment with a length of more than 1000 bp between the chloroplast and the mitochondria. This visualization provides a comprehensive view of the dynamics of gene transfer between these organelles.

In the mt genome of *H. sanguinea*, six sequence blocks highly similar to the cp genome were identified, indicating the existence of chloroplast-derived DNA migration events (**Figure 6**). The length of these migration fragments ranged from 2,295 bp to 3,390 bp. They mainly contain ribosomal RNA genes (such as *rrn16, rrn23, rrn4.5, rrn5*) and transfer RNA genes (such as *trnI-GAU, trnA-UGC, trnR-ACG*) (**Supplementary Table 7**). The alignment results show that the same cp region may appear many times in the mt genome, and integrate in both forward and reverse directions. For example, the ‘trnR-ACG* - rrn5 - rrn4.5 - rrn23*’ region corresponding to the chloroplast position of about 129–132 kb was detected twice in mitochondria (located at about 307.8-310.1 kb and 368.2-370.5 kb), and the integration direction was different. Most of these chloroplast-derived sequences are fragmented or partial genes, such as *trnR-ACG*, which is labeled as incomplete in mitochondria. These results suggest that chloroplast DNA may have been integrated into the mt genome through intracellular gene transfer events during the evolution of *H. sanguinea*, but most of these sequences may have lost their functions, reflecting the complex interaction and dynamic evolutionary history of plant organelle genomes.

### 3.6 Selective pressure analysis (Ka/Ks) in the mt genome

**Figure 7:**
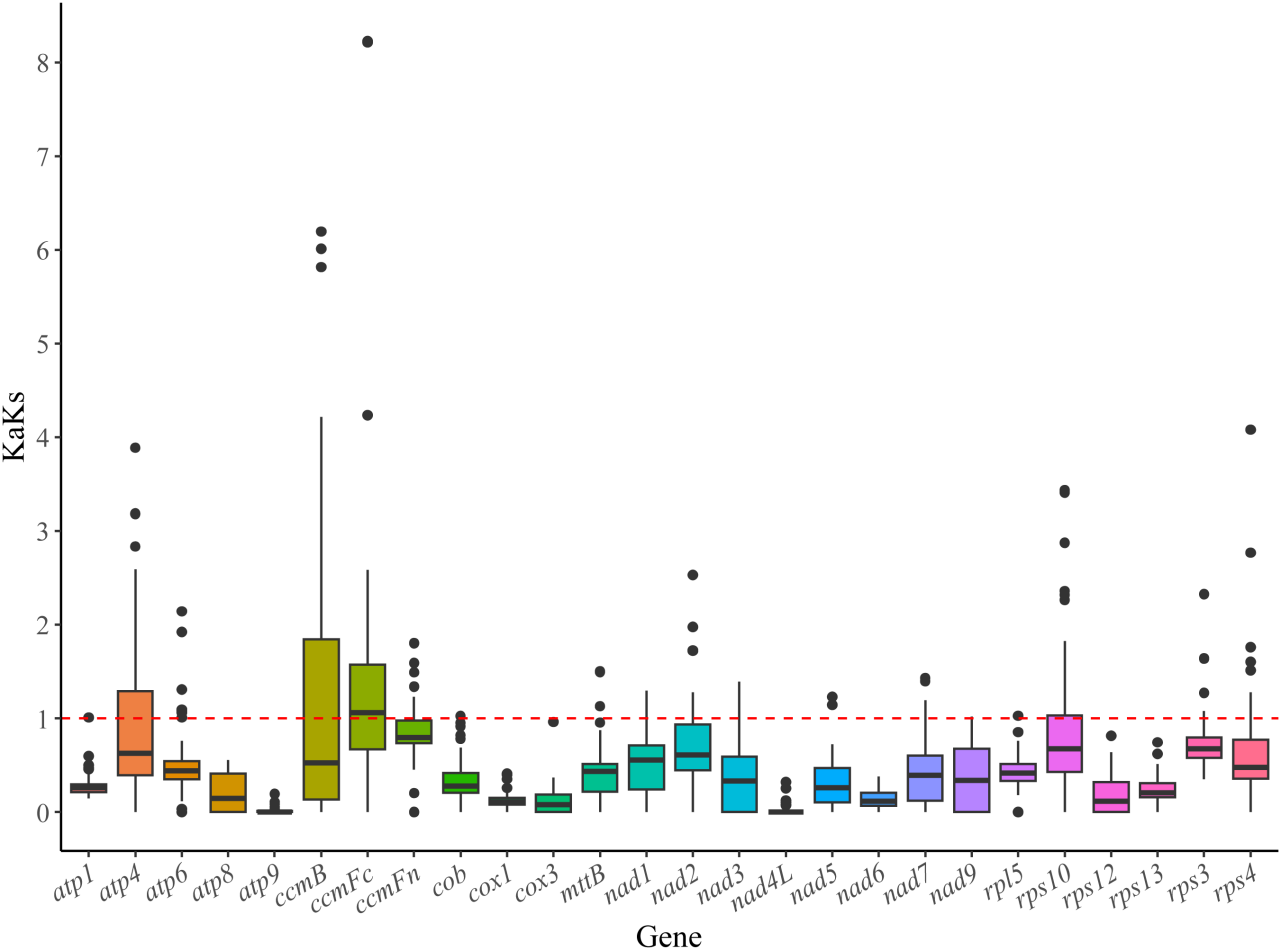
Ka/Ks values of 26 genes in the *H. sanguinea* mt genome. The red dashed line indicates a Ka/Ks value equal to 1.

Based on the analysis of the selection pressure (Ka/Ks) of the *H. sanguinea* mt genome, the results clearly revealed that the mitochondria-encoding genes were subjected to strong purifying selection pressure during evolution (**Figure 7**, **Supplementary Table 8**). The Ka/Ks ratios of most genes are significantly less than 1 (for example, close to 0 or less than 0.5), indicating that these genes are mainly subject to strong purifying selection during evolution. For example, the Ka/Ks ratios of *atp1, cob, cox1, nad1, nad3* and other genes are less than 0.5 among most species. This reflects the functional conservation of these genes, suggesting that these genes play an important role in maintaining the basic functions of mitochondria, especially the core protein-coding genes involved in the oxidative phosphorylation complex (such as *COX1, CYTB*, and the ND gene family), whose sequences are highly conserved to maintain the stability and efficiency of energy metabolism.

At the same time, the analysis also identified that a few genes (or specific gene regions) showed relatively high Ka/Ks values (such as some *ATP8* or ND genes), and the ratio was close to or slightly higher than 1, suggesting that these genes may have experienced relaxed selection constraints or local adaptive evolutionary events, which may be related to the energy adaptation, environmental response or functional differentiation of specific species. Overall, the mitochondrial genome exhibits extremely high functional conservation, and strong purifying selection is a central force that has dominated its molecular evolution, consistent with its key role as a cellular energy factory.

### 3.7 Nucleotide diversity analysis (Pi) in the mt genome

**Figure 8:**
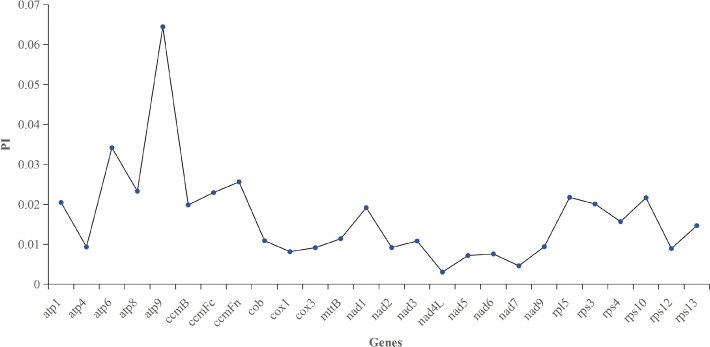
Nucleotide diversity analysis (Pi) of *H. sanguinea* mt genome genes.

According to the analysis of nucleotide diversity (Pi) of the mt genome, there are obvious differences in the level of genetic variation of each gene region. Among them, the *atp9* gene showed the highest nucleotide diversity (Pi = 0.06444), suggesting that this gene may have experienced more relaxed selection pressure or higher mutation accumulation. In contrast, the *nad4L* gene has the lowest Pi value (0.00305), indicating that its sequence is highly conserved and may be subject to strong functional constraints. The Pi values of most genes (such as *atp1, ccmB*, *cob, cox1, nad1*, etc.) are concentrated between 0.01 and 0.03, reflecting that the mt genome as a whole has a low level of genetic diversity (**Figure 8**, **Supplementary Table 9**).

Further observation showed that the core genes involved in energy metabolism (such as *cox1, nad5, nad6, nad7*, etc.) generally had low Pi values (all less than 0.01), indicating that these genes were subjected to strong purifying selection during evolution to maintain the stability of their key functions. However, some ATP synthase genes (such as *atp6, atp8*) and some ribosomal protein genes (such as *rpl5, rps3*) show relatively high genetic variation, which may be related to their functional plasticity or adaptive evolution. On the whole, the nucleotide diversity of the mitochondrial genome is consistent with its typical characteristics as an important organelle genome, that is, the core functional genes are highly conserved, while the auxiliary genes retain a certain degree of evolutionary flexibility.

### 3.8 Phylogenetic analysis

**Figure 9:**
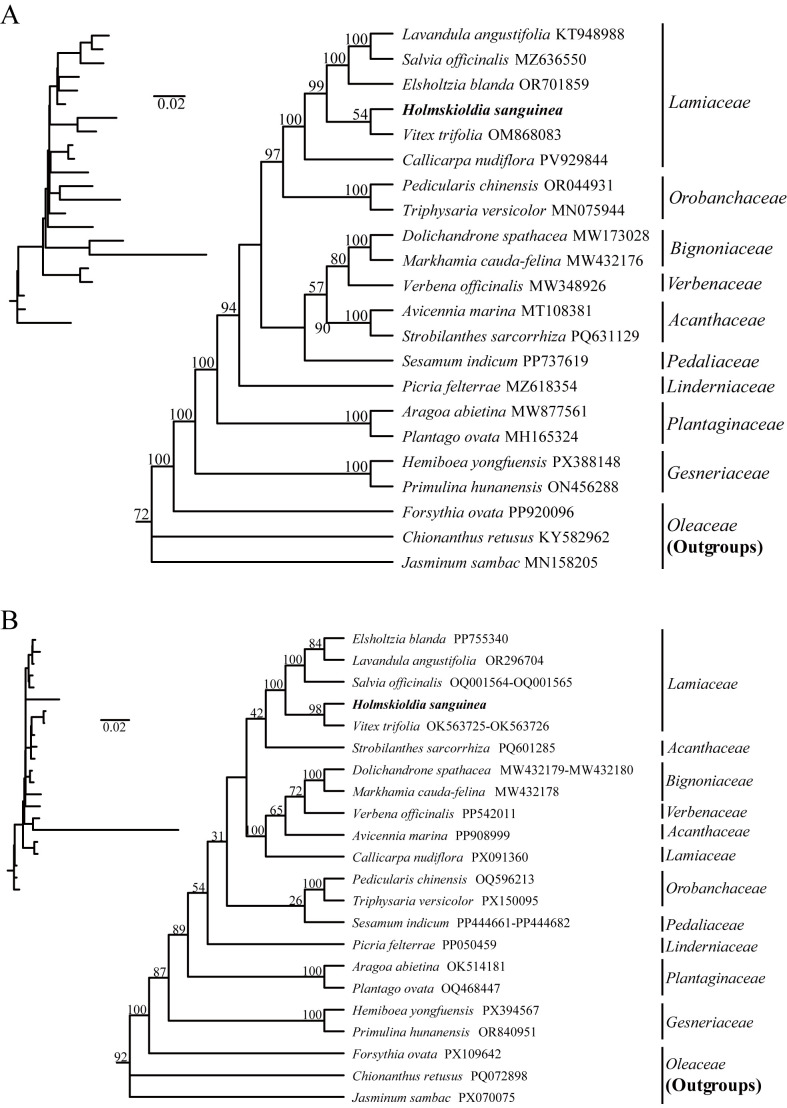
**(A)** Construct cp genome phylogenetic analysis of 23 species based on 49 core genes. **(B)** Construct mt genome phylogenetic analysis of 23 species based on 14 core genes.

The results of the cp phylogenetic tree based on 49 homologous single-copy protein-coding genes and the mt phylogenetic tree based on 14 homologous single-copy protein-coding genes are shown, scale bar of 0.02 substitutions per site (**Figure 9**, **Supplementary Table 10**). The analysis resolved most major clades, including Lamiaceae, Bignoniaceae, Acanthaceae, Verbenaceae, Orobanchaceae, Pedaliaceae, Linderniaceae, Plantaginaceae, Gesneriaceae and Oleaceae. Notably, *H. sanguinea* was placed within a well−supported clade (bootstrap value = 100%) that contained other Lamiaceae members, such as *L. angustifolia*, *S. officinalis*, *E. blanda*, *C. nudiflora* and *V. trifolia*. This clade was clearly distinct from adjacent families like Bignoniaceae and Verbenaceae.

Specifically, *H. sanguinea* grouped together with all other Lamiaceae taxa with a bootstrap support of 100%, confirming its robust phylogenetic affinity to this family. The close relationship was further evidenced by the short branch lengths within the Lamiaceae cluster, indicating relatively low sequence divergence among these species. Therefore, the result supports the classification of *H. sanguinea* within Lamiaceae. The phylogenetic position of the mt genome of the target species *H. sanguinea* within the order Lamiales was clarified.

### 3.9 Collinearity analysis

**Figure 10:**
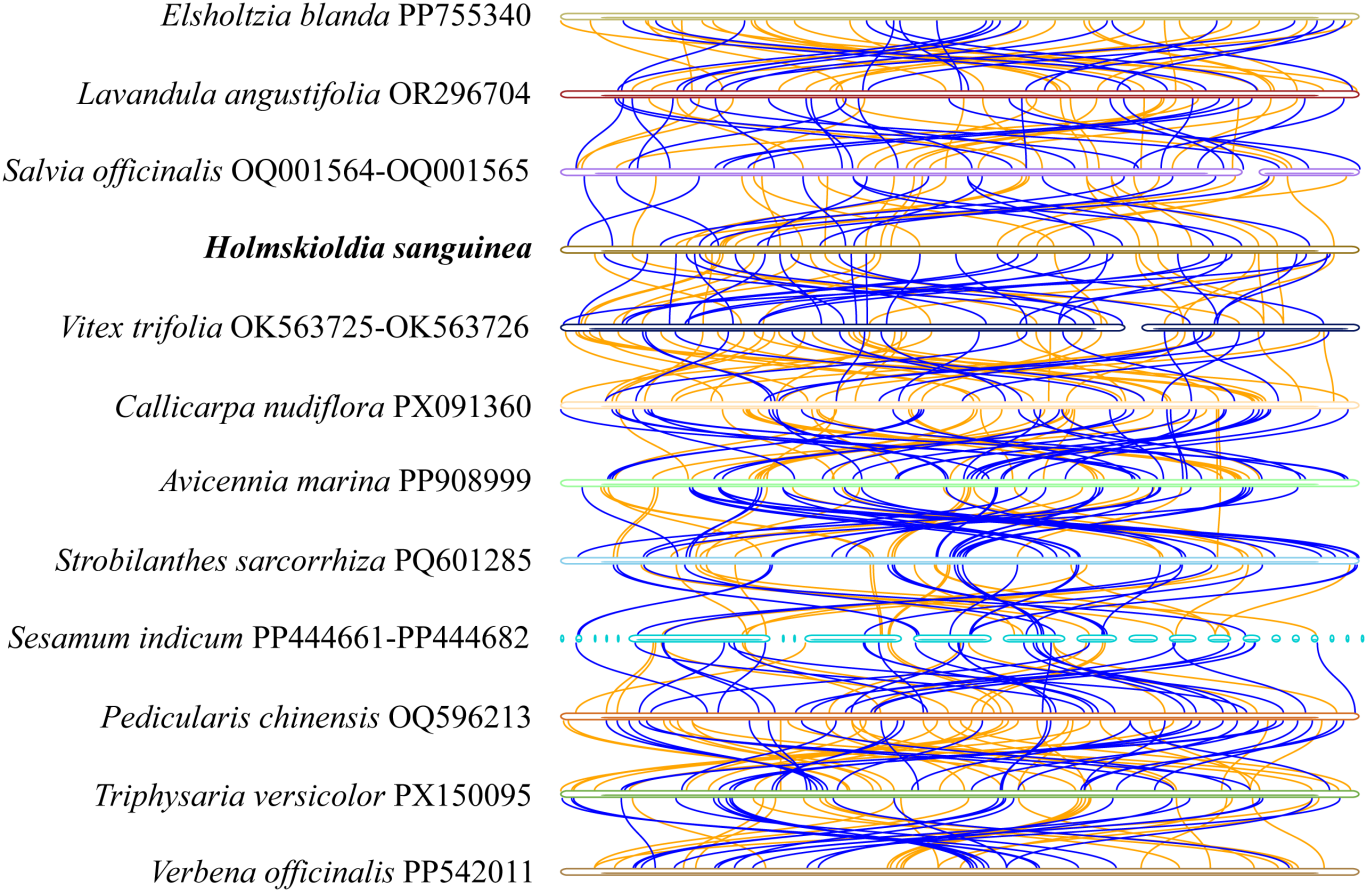
Collinearity analysis of 12 species. The regions connected by arcs in the figure represent regions with good homology, where the blue arcs indicate sequence reversal and the orange arcs indicate the forward direction of the sequence.

Collinearity analysis reveals the evolutionary association between species by exploring the conservation of homologous genetic sequences. The regions connected by arcs represent regions with good homology, in which the blue arcs represent reverse sequences and the orange arcs represent forward sequences (**Figure 10**). In addition, some regions that do not have collinear blocks are indicated to be unique to the species. Analysis of the mt genome syntenic blocks of 12 species revealed many homologous syntenic segments (**Supplementary Table 11**). Between *E.blanda* and *L.angustifolia*, *L. angustifolia* and *S. officinalis*, *S. officinalis* and *H. sanguinea*, *H. sanguinea* and *V. trifolia*, *V. trifolia* and *C. nudiflora*, *C. nudiflora* and *A. marina*, *A. marina* and *S. sarcorrhiza*, *S. sarcorrhiza* and *S. indicum*, *S. indicum* and *P. chinensis*, *P. chinensis* and *T. versicolor*, and *T. versicolor* and *V. officinalis*, 43 (112,227 bp), 45 (123,360 bp), 40 (114,983 bp), 50 (149,019 bp), 54 (173,044 bp), 64 (168,565 bp), 53 (83,131 bp), 41 (62,650 bp), 44 (99,227 bp), 60 (150,170 bp), and 45 (109,945 bp) mtDNA synteny blocks longer than 1000 bp were identified, respectively.

Based on pairwise BLAST alignments to identify mitochondrial collinear blocks with an alignment length ≥1000 bp and quantitatively analyzed the extent of genomic structural rearrangements among species. The results showed that the number of collinear blocks between adjacent species pairs ranged from 40 to 64 (**Supplementary Table 12**). Among them, *C. nudiflora* and *A. marina* exhibited the highest number of collinear fragments (64), indicating the most frequent breakage and rearrangement events in their mt genomes; in contrast, *S. officinalis* and *H. sanguinea* had the fewest collinear blocks (40), reflecting relatively higher collinearity conservation. Across all aligned pairs, the average length of a single collinear fragment ranged from 1528.05 to 3204.52 bp, and homologous fragments were shorter and more fragmented between species from different families, further reflecting the extensive structural rearrangements that occurred in mt genomes during evolution.

The abundance of mitochondrial repetitive sequences in Lamiaceae and other taxa was compared using the Mann–Whitney U test (Wilcoxon rank-sum test). The results showed that the average proportion of total repeats was 5.52% in Lamiaceae and 5.94% in other taxa, with no significant difference between the two groups (P > 0.05). The average proportion of long repeats (≥100 bp) was 3.54% in Lamiaceae and 3.03% in other taxa, and again no statistically significant difference was detected between groups (P > 0.05) (**Supplementary Table 12**).

By integrating repeat annotation and collinearity alignment results, we further analyzed their spatial association patterns. The results revealed that the vast majority of mt genome rearrangement breakpoints were adjacent to long repeat regions, with the break intervals overlapping or adjacent to long repeat elements, indicating a significant spatial association between repeats and genomic fragmentation and fragment rearrangement. In species pairs with the highest degree of collinearity fragmentation, the density of long repeats around breakpoints was also markedly higher, further demonstrating that repeat distribution is closely associated with the occurrence of mt rearrangements. These findings confirm that long repeats are not randomly distributed but are highly coupled with mt rearrangement breakpoints. Homologous recombination mediated by long repeats is the direct cause of homologous fragment breakage, positional rearrangement, and the collapse of collinearity.

### 3.10 Gene deletion

**Figure 11:**
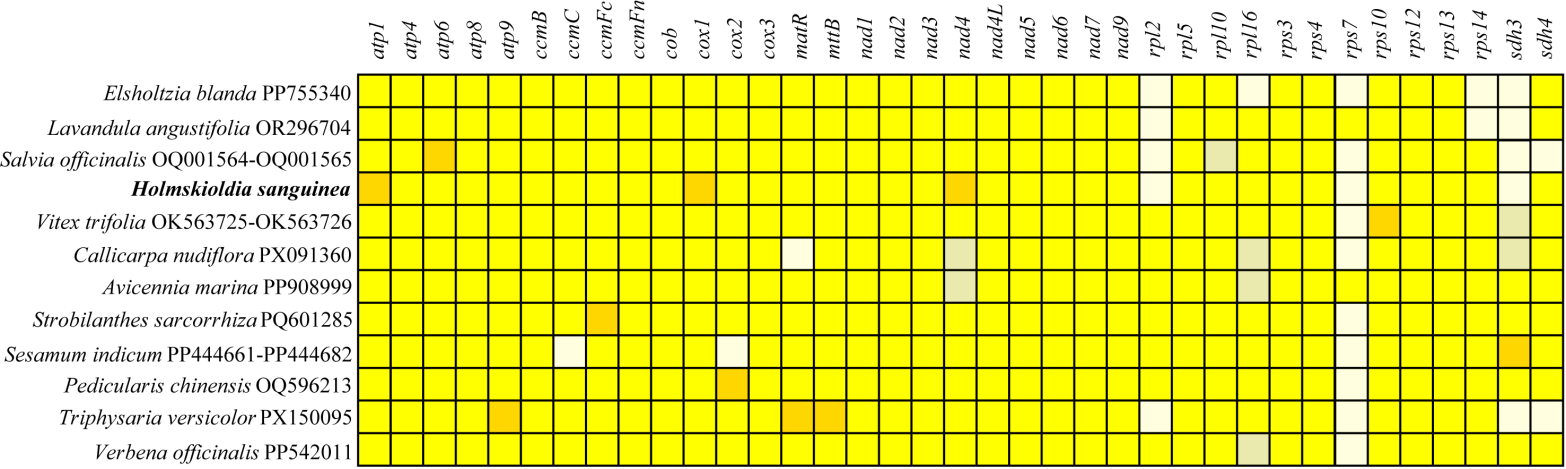
Analysis of gene deletion. Note: Yellow in the figure represents genes present in the mt genome, white represents genes lost in the mt genome, and gray color represents pseudogene and orange represents copy genes.

The distribution of core PCGs in the mitochondrial genomes of 12 species was compared. As shown in **Figure 11**, most PCGs are conserved, especially the genes for ATP synthase, transport membrane protein, and NADH dehydrogenase. In contrast, the ribosomal protein and succinate dehydrogenase genes were more variable. It is understandable that *rpl2, rpl10, rpl16, rps7, rps14, sdh3* and *sdh4* genes were lost in part of the mitochondrial genomes, while *rps1, rps2, rps11, rps19* and *rpl6* genes were all lost in all 12 plant mitochondrial genomes, as ribosomal protein genes are often lost or transferred to the nucleus during the evolution of angiosperm mt genomes.

Although there are different degrees of gene deletion or pseudogenization in all species, *H. sanguinea* still showed unique gene content characteristics: there are two copies of *atp1, cox1* and *nad4* genes, suggesting that gene duplication events might have occurred. At the same time, *rps7, rpl2* and *sdh3* genes were completely deleted in this species, while most of the other genes remained as single-copy intact. This result suggests that *H. sanguinea* experienced both a loss of function of specific genes and an increase in the copy number of certain genes through gene duplication during evolution. On the whole, the distribution of gene deletion and pseudogenization is uneven among different species; for example, *matR* is deleted, and *nad4* and *rpl16* are pseudogenes in *C. nudiflora*; *ccmC* and *cox2* are deleted in *S. indicum*; *T. versicolor* has duplications or deletions in multiple genes. These variations together reflect the dynamic evolutionary history of the mt genome structure of these 12 species.

## 4 Discussion

*H. sanguinea* is an important ornamental, and the complete analysis of its mt genome not only provides key molecular evidence for clarifying the phylogenetic status of the species, but also opens up a new window for understanding the evolutionary law, structural variation and cooperative adaptation mechanism with the nuclear genome of plant mitochondria, a dynamic organelle genome. In this study, the first mt genome of *H. sanguinea* was successfully assembled and analyzed by multi-dimensional comparative genomics. The results show that the genome exhibits the typical complexity of plant mitochondria, including large genome size, abundant repetitive sequences, significant codon usage bias, extensive RNA editing events, and frequent gene transfer between organelles. Phylogenetic analysis clarified its taxonomic position within Lamiaceae, while synteny and gene content analyses revealed drastic genome rearrangements and a dynamic evolutionary history. In the following, we will combine the results of this study with the frontier discoveries in recent years to discuss the characteristics of the mt genome and its evolutionary significance.

### 4.1 Genome structure characteristics, dynamics and evolutionary driving force

Plant mt genomes frequently exist as a dynamic population of subgenomic molecules rather than a single master circle, and such multi-branched graphs often reflect alternative conformations generated by repeat-mediated recombination (Xia et al., 2023). *H. sanguinea* mt genome exhibited a multi-branched conformation with a total length of 405,461 bp and a GC content of 46.00%, which is slightly higher than many reported mt genomes of angiosperms (usually 43–45%). Higher GC content may enhance the physical stability of the DNA double helix by increasing the number of hydrogen bonds (Chen and Skylaris, 2021) and may affect gene expression regulation. Compared with the chloroplast genome, the mt genome is significantly enlarged, which is mainly attributed to the expansion of non-coding regions, the accumulation of repetitive sequences, and the integration of foreign sequences (Wang et al., 2024a). In this study, six distinct chloroplast-derived sequence blocks were found, with a considerable total length, confirming that intracellular gene transfer (IGT) is one of the key evolutionary forces shaping the plant mitochondrial genome (Gualberto et al., 2014). Notably, the same chloroplast region was integrated multiple times in mitochondria with different orientations and locations, strongly suggesting a central role of repeat-mediated homologous recombination in genome remodeling (Sloan et al., 2018). The GC content of CDS (42.25%) was lower than that of tRNA (51.57%) and rRNA (52.03%) genes, reflecting the difference in selection pressure in different functional regions.

### 4.2 Repeat sequences

Repeat sequences are central to the understanding of size variation and structural rearrangements in plant mitochondrial genomes. In this study, 79 SSRs and 550 pairs of discrete repeats with length ≥ 30 bp were identified in the mitochondria of *H. sanguinea*, of which short repeats of 30-69 bp accounted for the vast majority (96.2%). Much evidence shows that such short interspersed repeats are the main factors driving the frequent recombination and conformational diversification of many plant mt genomes (Wu et al., 2015; Liu et al., 2021). SSRs are most abundant in tetranucleotide repeats (AAAG/CTTT), and these highly variable microsatellite sequences provide valuable resources for the development of molecular markers for species identification and population genetics (Čechová et al., 2018). The abundance and distribution of repetitive sequences are directly related to the recombination frequency and evolution rate of the genome, providing a material basis for its rapid structural evolution, and may also contribute to adaptive evolution by affecting the expression of adjacent genes (Christensen, 2013; Wynn and Christensen, 2019). Compared with *M. ruthenica* (containing 148 discrete repeats), The mt genome of *H. sanguinea* has more repeat pairs (550 pairs), suggesting that it may have higher genomic structural plasticity and recombination potential.

### 4.3 Codon usage preference

Codon usage bias analysis of 28 core protein-coding genes revealed a strong preference for synonymous codons ending in A or U in *H. sanguinea* mitochondria, a common feature of organelle genomes in terrestrial plants. This preference is often attributed to a combination of mutational pressure (AT-biased mutation) and natural selection aimed at optimizing translation efficiency or accuracy (Jia and Higgs, 2008). Alanine codon GCU had the highest relative synonymous codon usage (RSCU) value (1.57), while the stop codon UAA was significantly preferred, suggesting that there may be a specialized translation regulation mechanism for specific amino acids and termination process. In contrast to legumes such as *M. ruthenica*, *H. sanguinea* did not show a specific preference for UGA stop codons, suggesting that codon usage patterns may be family and genus-specific. Recent studies have shown that codon bias may be tightly coupled with tRNA abundance, translation elongation rate and protein folding efficiency, thus affecting the physiological response of cells under stress conditions (Wu et al., 2019; Yang et al., 2024). While it is tempting to speculate that this codon usage pattern might facilitate translational flexibility under stress conditions, direct experimental evidence is needed to test this hypothesis.

### 4.4 RNA editing

We predicted 565 C-to-U RNA editing sites in 34 PCGs, and most of the editing events (80.71%) resulted in the change of amino acids from hydrophilic to hydrophobic. This trend is highly conserved in plant mitochondria, and its core function is to correct genomic CDS and produce functional hydrophobic transmembrane proteins, which is essential for the correct assembly and function of respiratory chain complexes located in the inner mitochondrial membrane (Giegé and Brennicke, 1999; Takenaka et al., 2013). The number of editing sites varies greatly among different genes (*nad4* is the most and *atp9* is the least), reflecting the differences in gene functional constraints and evolutionary history. In recent years, more and more studies have shown that RNA editing is an important post-transcriptional regulatory layer in plant response to abiotic stress. In this study, we found that there were wide-ranging RNA editing events in the mitochondria of *H. sanguinea*, and their site distribution and frequency were significantly different among different genes, which may be related to its potential ability to cope with environmental changes. Recent studies have provided direct evidence for this view: for example, in rice, the *PPR* protein *PPR767* located in mitochondria not only affects the activity of mitochondrial complex I by specifically editing specific sites of *nad1, nad3* and other genes, but also significantly affects plant drought resistance by regulating reactive oxygen species (ROS) content (Peng et al., 2025). Similarly, in wheat, comparative studies of drought-tolerant and drought-sensitive cultivars have found significant differences in the RNA editing patterns of the *NAD9* gene, and these editing events directly alter the structure of the *NAD9* protein, which is considered to be an important molecular basis for differences in drought resistance in wheat (Mohamed et al., 2024). Together, these cases suggest that site-specific RNA editing, mediated by editing factors encoded by nuclear genes (such as *PPR* proteins), is a precise regulatory mechanism for mitochondrial function to adapt to environmental stress. Although we have not experimentally tested the role of editing in stress responses, recent studies in rice and wheat (Peng et al., 2025; Mohamed et al., 2024) have demonstrated such links, suggesting a possible direction for future research in *H. sanguinea*.

### 4.5 Selection pressure and nucleotide diversity

Ka/Ks analysis showed that most of the mt genes were under strong purifying selection (Ka/Ks < 1), especially the genes encoding the core subunits of the respiratory chain (such as *cox1, cob, nad1*), which highlighted the irreplaceability of these genes in maintaining basic energy metabolism (Jacoby et al., 2012). This is consistent with the general slow evolution of plant mt genes (Sloan et al., 2012). However, nucleotide diversity (Pi) analysis revealed significant differences in the level of variation among different genes. The *atp9* gene showed the highest Pi value (0.06444), while *nad4L* showed the lowest Pi value (0.00305). Highly variable genes (such as *atp9, atp6*) may be under relatively relaxed functional constraints or undergoing adaptive evolution. This “conservative at the core, variable at the periphery” pattern, similar to the pattern observed in *M. ruthenica* (Tian et al., 2025), is typical of biological systems that strike a balance between maintaining basic functional stability and acquiring adaptive innovation (Takezawa and Minami, 2004). Comparison of the selection signals of these variable genes between Verbena and related species is expected to reveal the potential molecular adaptation mechanism of species differentiation in Verbenaceae.

### 4.6 Phylogeny, synteny and changes in gene content

Phylogenetic analysis using 14 mt PCGs placed *H. sanguinea* firmly within Lamiaceae, forming a well-supported clade (bootstrap = 100%) with *L. angustifolia*, *S. officinalis*, *E. blanda*, *C. nudiflora* and *V. trifolia* (**Figure 9B**). This result resolves the long-standing controversy regarding the family assignment of *Holmskioldia* and corroborates recent molecular studies that suggested a Lamiaceae affinity (Li et al., 2016; Lee and Kim, 2020). The short branch lengths within the Lamiaceae clade indicate relatively low sequence divergence among these species, reflecting their shared evolutionary history. It should be noted that Lamiales, as a highly species-diverse order within angiosperms, has long suffered from unclear clustering in its family-level phylogenetic relationships (Stettler et al., 2021). Comparative analyses based on complete plastid genomes and nuclear genomes are gradually clarifying these relationships (Dong et al., 2022).

Collinearity analysis revealed extensive genome rearrangements among the compared Lamiales species. A large number of synteny blocks (>1000 bp) were identified between *H. sanguinea* and other Lamiaceae members, but the order of these blocks was highly inconsistent, indicating frequent recombination events. This structural plasticity is typical of plant mt genomes and may have contributed to the diversification of Lamiaceae.

Comparison of gene content showed that the loss of ribosomal protein genes *rps7*, *rpl2* and *sdh3* is widespread in the compared Lamiaceae species. The loss of ribosomal protein genes, or their transfer to the nucleus, is a general trend in the evolution of angiosperm mt genomes (Adams et al., 2002). While the duplication of *atp1*, *cox1* and *nad4* appears to be a lineage-specific feature of *H. sanguinea* (**Supplementary Table 11**). These gene content variations, together with the phylogenetic placement, provide a robust framework for understanding the evolution of Lamiaceae mitochondrial genomes. The duplication of the *atp1, cox1*, and *nad4* genes may enhance mt energy production, which macroscopically manifests as strong environmental adaptability and rapid growth of *H. sanguinea*, making it suitable for slope ecological restoration.

## 5 Conclusion

In summary, the *H. sanguinea* mt genome shows a typical but unique plant organelle genome blueprint: it has a complex structure, active repetitive sequences, obvious translational and post-transcriptional regulatory features, and retains the potential for local adaptive variation in the context of strong purifying selection. Phylogenetic analysis based on *H. sanguinea* mt genome clarified its taxonomic position within Lamiaceae. This study provides key data for the conservation, development and utilization of genetic resources of *H. sanguinea*.

## Data Availability

The datasets presented in this study can be found in online repositories. The names of the repository/repositories and accession number(s) can be found below: https://www.ncbi.nlm.nih.gov/, PX778768; https://www.ncbi.nlm.nih.gov/, PX778767; https://www.ncbi.nlm.nih.gov/, PRJNA1393605; https://www.ncbi.nlm.nih.gov/, SAMN54307066; https://www.ncbi.nlm.nih.gov/, SRR36601807.

## References

[B1] Adams K. L. Qiu Y. L. Stoutemyer M. Palmer J. D. (2002). Punctuated evolution of mitochondrial gene content: high and variable rates of mitochondrial gene loss and transfer to the nucleus during angiosperm evolution. Proc. Natl. Acad. Sci. U.S.A. 99, 9905–9912. doi: 10.1073/pnas.042694899 12119382 PMC126597

[B2] Altschul S. F. Gish W. Miller W. Myers E. W. Lipman D. J. (1990). Basic local alignment search tool. J. Mol. Biol. 215, 403–410. doi: 10.1016/S0022-2836(05)80360-2 2231712

[B3] Alverson A. J. Wei X. Rice D. W. Stern D. B. Barry K. Palmer J. D. (2010). Insights into the evolution of mitochondrial genome size from complete sequences of Citrullus lanatus and Cucurbita pepo (Cucurbitaceae). Mol. Biol. Evol. 27, 1436–1448. doi: 10.1093/molbev/msq029 20118192 PMC2877997

[B4] Arseneau J. Steeves R. Laflamme M. (2017). Modified low-salt CTAB extraction of high-quality DNA from contaminant-rich tissues. Mol. Ecol. Resour. 17, 686–693. doi: 10.1111/1755-0998.12616 27768249

[B5] Atkins S. (1996). Holmskioldia sanguinea Labiatae, formerly Verbenaceae. Bot. Mag 293, 79–81. doi: 10.1111/j.1467-8748.1996.tb00545.x 40046247

[B6] Beck N. Lang B. F. (2010). MFANNOT: a web server for annotating organellar genomes. Nucleic Acids Res. 38, W35–W40. doi: 10.1093/nar/gkq427 20525785

[B7] Benson G. (1999). Tandem repeats finder: a program to analyze DNA sequences. Nucleic Acids Res. 27, 573–580. doi: 10.1093/nar/27.2.573 9862982 PMC148217

[B8] Bi C. Shen W. Wang D. Liu C. (2024). PMAT: an efficient plant mitochondrial genome assembler using PacBio HiFi reads. Bioinformatics 40, btae119. doi: 10.1093/bioinformatics/btae119 38426351 PMC10955254

[B9] Čechová J. Lýsek J. Bartas M. Brázda V. (2018). Complex analyses of inverted repeats in mitochondrial genomes revealed their importance and variability. Bioinformatics 34, 1081–1085. doi: 10.1093/bioinformatics/btx729 29126205 PMC6030915

[B10] Chaudhuri P. K. Srivastava R. Kumar S. Kumar S. (2004). Phytotoxic and antimicrobial constituents of Bacopa monnieri and Holmskioldia sanguinea. Phytother Res. 18, 114–117. doi: 10.1002/ptr.1278 15022161

[B11] Chen C. Chen H. Zhang Y. Thomas H. R. Frank M. H. He Y. . (2020). TBtools: an integrative toolkit developed for interactive analyses of big biological data. Mol. Plant 13, 1194–1202. doi: 10.1016/j.molp.2020.06.009 32585190

[B12] Chen H. Skylaris C. K. (2021). Analysis of DNA interactions and GC content with energy decomposition in large-scale quantum mechanical calculations. Phys. Chem. Chem. Phys. 23, 8891–8899. doi: 10.1039/d0cp06630c 33876048

[B13] Christensen A. C. (2013). Plant mitochondrial genome evolution can be explained by DNA repair mechanisms. Genome Biol. Evol. 5, 1079–1086. doi: 10.1093/gbe/evt069 23645599 PMC3698917

[B14] Dong X. Mkala E. M. Mutinda E. S. Yang J. X. Wanga V. O. Oulo M. A. . (2022). Taxonomy, comparative genomics of Mullein (Verbascum, Scrophulariaceae), with implications for the evolution of Verbascum and Lamiales. BMC Genomics 23, 566. doi: 10.1186/s12864-022-08799-9 35941527 PMC9358837

[B15] Fonseca R. R. D. Johnson W. E. O'Brien S. J. . (2008). The adaptive evolution of the mammalian mitochondrial genome. BMC Genomics 9, 119. doi: 10.1186/1471-2164-9-119 18318906 PMC2375446

[B16] Giegé P. Brennicke A. (1999). RNA editing in Arabidopsis mitochondria effects 441 C to U changes in ORFs. Proc. Natl. Acad. Sci. U.S.A. 96, 15324–15329. doi: 10.1073/pnas.96.26.15324 10611383 PMC24818

[B17] Greiner S. Lehwark P. Bock R. (2019). OrganellarGenomeDRAW (OGDRAW) version 1.3.1: expanded toolkit for the graphical visualization of organellar genomes. Nucleic Acids Res. 47, W59–W64. doi: 10.1093/nar/gkz238 30949694 PMC6602502

[B18] Gualberto J. M. Mileshina D. Wallet C. Niazi A. K. Weber-Lotfi F. Dietrich A. (2014). The plant mitochondrial genome: dynamics and maintenance. Biochimie 100, 107–120. doi: 10.1016/j.biochi.2013.09.016 24075874

[B19] Jacoby R. P. Li L. Huang S. Pong Lee C. Millar A. H. Taylor N. L. (2012). Mitochondrial composition, function and stress response in plants. J. Integr. Plant Biol. 54, 887–906. doi: 10.1111/j.1744-7909.2012.01177.x 23046139

[B20] Jia W. Higgs P. G. (2008). Codon usage in mitochondrial genomes: distinguishing context-dependent mutation from translational selection. Mol. Biol. Evol. 25, 339–351. doi: 10.1093/molbev/msm259 18048402

[B21] Kalyaanamoorthy S. Minh B. Q. Wong T. K. F. von Haeseler A. Jermiin L. S. (2017). ModelFinder: fast model selection for accurate phylogenetic estimates. Nat. Methods 14, 587–589. doi: 10.1038/nmeth.4285 28481363 PMC5453245

[B22] Kong J. L. Wang J. Nie L. Y. Tembrock L. R. Zou C. S. Kan S. L. . (2025). Evolutionary dynamics of mitochondrial genomes and intracellular transfers among diploid and allopolyploid cotton species. BMC Biol. 23, 9. doi: 10.1186/s12915-025-02115-z 39794789 PMC11720916

[B23] Kurtz S. Choudhuri J. V. Ohlebusch E. Schleiermacher C. Stoye J. Giegerich R. (2001). REPuter: the manifold applications of repeat analysis on a genomic scale. Nucleic Acids Res. 29, 4633–4642. doi: 10.1093/nar/29.22.4633 11713313 PMC92531

[B24] Lee Y. Kim S. (2020). The complete chloroplast genome sequence of Holmskioldia sanguinea Retz. an ornamental plant of Lamiaceae. Mitochondrial DNA B. Resour. 27, 895–896. doi: 10.1080/23802359.2020.1717392 33366801 PMC7748846

[B25] Li H. (2018). Minimap2: pairwise alignment for nucleotide sequences. Bioinformatics 34, 3094–3100. doi: 10.1093/bioinformatics/bty191 29750242 PMC6137996

[B26] Li B. Cantino P. D. Olmstead R. G. Bramley G. L. Xiang C. L. Ma Z. H. . (2016). A large-scale chloroplast phylogeny of the Lamiaceae sheds new light on its subfamilial classification. Sci. Rep. 6, 34343. doi: 10.1038/srep34343 27748362 PMC5066227

[B27] Lin S. S. Liao L. He W. Hu W. S. Sun Y. J. (2017). Ecological landscape construction practice for Holmskioldia sanguinea, an excellent plant food source for birds. Guangdong Gardens Magazine 1, 71–75. doi: 10.3969/j.issn.1671-2641.2017.01.015

[B28] Liu H. Yu J. Yu X. Zhang D. Chang H. Li W. . (2021). Structural variation of mitochondrial genomes sheds light on evolutionary history of soybeans. Plant J. 108, 1456–1472. doi: 10.1111/tpj.15522 34587339

[B29] Liu S. Zhang Y. Li L. Huang D. Qin Y. (2025). Assembly and comparative analysis of the complete mitochondrial genome of Indocalamus longiauritus. Front. Plant Sci. 16, 1599464. doi: 10.3389/fpls.2025.1599464 40584851 PMC12202455

[B30] Mohamed N. G. Ramadan A. M. Amer M. Morsy Y. Mohamed R. A. Said O. A. M. . (2024). RNA editing-induced structural and functional adaptations of NAD9 in Triticum aestivum under drought stress. Front. Plant Sci. 15, 1490288. doi: 10.3389/fpls.2024.1490288 39600896 PMC11590480

[B31] Peng L. Xiao H. Xu Y. Huang Z. Yang X. Lv C. . (2025). The pentatricopeptide repeat protein PPR767 modulates plant architecture and drought resistance in rice. Plant Physiol. 199, kiaf187. doi: 10.1093/plphys/kiaf187 40331370

[B32] Ronquist F. Teslenko M. van der Mark P. Ayres D. L. Darling A. Höhna S. . (2012). MrBayes 3.2: efficient Bayesian phylogenetic inference and model choice across a large model space. Syst. Biol. 61, 539–542. doi: 10.1093/sysbio/sys029 22357727 PMC3329765

[B33] Rozas J. Ferrer-Mata A. Carlos Sanchez-DelBarrio J. Guirao-Rico S. Librado P. Ramos-Onsins S. E. . (2017). DnaSP 6: DNA sequence polymorphism analysis of large data sets. Mol. Biol. Evol. 34, 3299–3302. doi: 10.1093/molbev/msx248 29029172

[B34] Ryding O. (1995). Pericarp structure and phylogeny of the Lamiaceae-Verbenaceae complex. Plant Syst. Evol. 198, 101–141. doi: 10.1007/BF00985109 30311153

[B35] Sloan D. B. Alverson A. J. Chuckalovcak J. P. Wu M. McCauley D. E. Palmer J. D. . (2012). Rapid evolution of enormous, multichromosomal genomes in flowering plant mitochondria with exceptionally high mutation rates. PloS Biol. 10, e1001241. doi: 10.1371/journal.pbio.1001241 22272183 PMC3260318

[B36] Sloan D. B. Wu Z. Sharbrough J. (2018). Correction of persistent errors in Arabidopsis reference mitochondrial genomes. Plant Cell 30, 525–527. doi: 10.1105/tpc.18.00024 29519893 PMC5894837

[B37] Stamatakis A. (2006). RAxML-VI-HPC: maximum likelihood-based phylogenetic analyses with thousands of taxa and mixed models. Bioinformatics 22, 2688–2690. doi: 10.1093/bioinformatics/btl446 16928733

[B38] Stettler J. M. Stevens M. R. Meservey L. M. Crump W. W. Grow J. D. Porter S. J. . (2021). Improving phylogenetic resolution of the Lamiales using the complete plastome sequences of six Penstemon species. PloS One 16, e0261143. doi: 10.1371/journal.pone.0261143 34910738 PMC8673674

[B39] Takenaka M. Zehrmann A. Verbitskiy D. Härtel B. Brennicke A. (2013). RNA editing in plants and its evolution. Annu. Rev. Genet. 47, 335–352. doi: 10.1146/annurev-genet-111212-133519 24274753

[B40] Takezawa D. Minami A. (2004). Calmodulin-binding proteins in bryophytes: identification of abscisic acid-, cold-, and osmotic stress-induced genes encoding novel membrane-bound transporter-like proteins. Biochem. Biophys. Res. Commun. 317, 428–436. doi: 10.1016/j.bbrc.2004.03.052 15063776

[B41] Talavera G. Castresana J. (2007). Improvement of phylogenies after removing divergent and ambiguously aligned blocks from protein sequence alignments. Syst. Biol. 56, 564–577. doi: 10.1080/10635150701472164 17654362

[B42] Thiel T. Michalek W. Varshney R. K. Graner A. (2003). Exploiting EST databases for the development and characterization of gene-derived SSR-markers in barley (Hordeum vulgare L.). Theor. Appl. Genet. 106, 411–420. doi: 10.1007/s00122-002-1031-0 12589540

[B43] Tian Y. Wu Z. Tian C. Yang Y. Li Z. (2025). Phylogenetic classification and genetic insights from the complete mitochondrial genome of Medicago ruthenica. Front. Plant Sci. 16, 1648505. doi: 10.3389/fpls.2025.1648505 40978774 PMC12446361

[B44] Wang J. Kan S. Liao X. Zhou J. Tembrock L. R. Daniell H. . (2024a). Plant organellar genomes: much done, much more to do. Trends Plant Sci. 29, 754–769. doi: 10.1016/j.tplants.2023.12.014 38220520

[B45] Wang J. Zou Y. Mower J. P. Wayne R. Wu Z. Q. (2024b). Rethinking the mutation hypotheses of plant organellar DNA. Genomics Commun. 1, e003. doi: 10.48130/gcomm-0024-0003

[B46] Wick R. R. Schultz M. B. Zobel J. Holt K. E. (2015). Bandage: interactive visualization of de novo genome assemblies. Bioinformatics 31, 3350–3352. doi: 10.1093/bioinformatics/btv383 26099265 PMC4595904

[B47] Wickham H. (2009). Ggplot2: Elegant Graphics for Data Analysis (New York: Springer).

[B48] Wu Z. Cuthbert J. M. Taylor D. R. Sloan D. B. (2015). The massive mitochondrial genome of the angiosperm Silene noctiflora is evolving by gain or loss of entire chromosomes. Proc. Natl. Acad. Sci. U.S.A. 112, 10185–10191. doi: 10.1073/pnas.1421397112 25944937 PMC4547255

[B49] Wu C. Zinshteyn B. Wehner K. Green R. (2019). High-resolution ribosome profiling defines discrete ribosome elongation states and translational regulation during cellular stress. Mol. Cell 73, 959–970. doi: 10.1016/j.molcel.2018.12.009 30686592 PMC6411040

[B50] Wynn E. L. Christensen A. C. (2019). Repeats of unusual size in plant mitochondrial genomes: identification, incidence and evolution. G3 Genes Genomes Genet. 9, 549–559. doi: 10.1534/g3.118.200948 30563833 PMC6385970

[B51] Xia C. Li J. Zuo Y. . (2023). Complete mitochondrial genome of Thuja sutchuenensis and its implications on evolutionary analysis of complex mitogenome architecture in Cupressaceae. BMC Plant Biol. 23, 84. doi: 10.1186/s12870-023-04054-9 36750935 PMC9903464

[B52] Xu J. X. Fu W. M. Zhang J. (2023). Drought resistance of 5 species of slope greening plants in South China. Guangdong Gardens Magazine 45, 85–89. doi: 10.12233/j.gdyl.2023.03.015

[B53] Yang Y. Wang X. Shi Z. (2024). Comparative study on codon usage patterns across chloroplast genomes of eighteen Taraxacum species. Horticulturae 10, 492. doi: 10.3390/horticulturae10050492 30654563

[B54] Zervas A. Petersen G. Seberg O. (2019). Mitochondrial genome evolution in parasitic plants. BMC Evol. Biol. 19, 87. doi: 10.1186/s12862-019-1401-8 30961535 PMC6454704

[B55] Zhang D. Gao F. Jakovlić I. Zou H. Zhang J. Li W. X. . (2020). PhyloSuite: an integrated and scalable desktop platform for streamlined molecular sequence data management and evolutionary phylogenetics studies. Mol. Ecol. Resour. 20, 348–355. doi: 10.1111/1755-0998.13096 31599058

[B56] Zhang Z. Li J. Zhao X. Q. Wang J. Wong G. K. S. Yu J. (2006). KaKs_Calculator: calculating Ka and Ks through model selection and model averaging. Genom. Proteom Bioinf 4, 259–263. doi: 10.1016/S1672-0229(07)60007-2 17531802 PMC5054075

[B57] Zhang H. Meltzer P. Davis S. (2013). RCircos: an R package for Circos 2D track plots. BMC Bioinf 14, 244. doi: 10.1186/1471-2105-14-244 23937229 PMC3765848

