# Supplementary figures and images for: Assembly and comparative analysis of the complete mitochondrial genome of Holmskioldia sanguinea
Source: Front Plant Sci. 2026 Jun 16;17:1867148. doi: 10.3389/fpls.2026.1867148 (PMC13314627; doi:10.3389/fpls.2026.1867148)

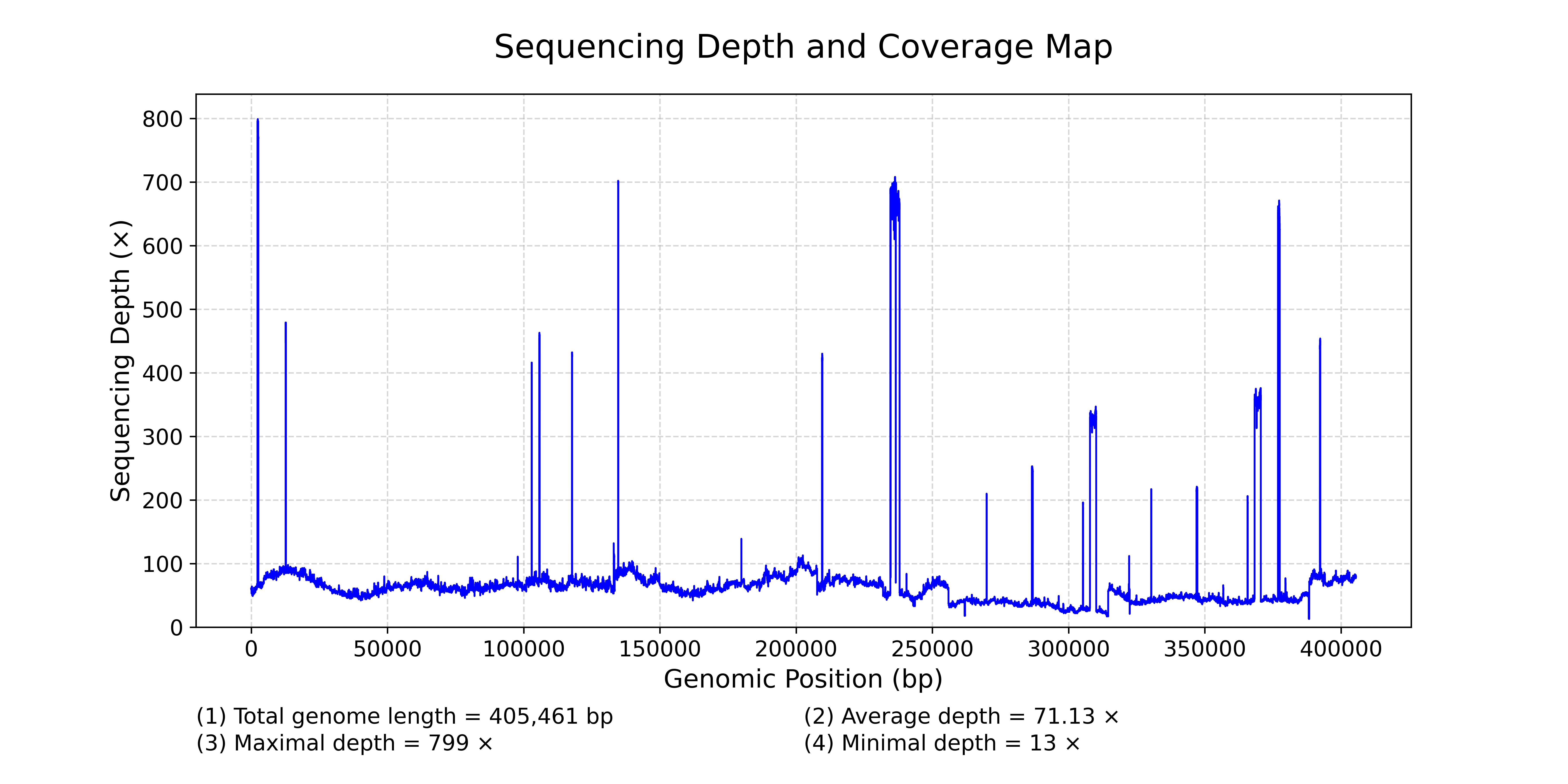

Supplement: Supplementary file 2 [file Image1.jpeg]
